# Airway wall thickness on HRCT scans decreases with age and increases with smoking

**DOI:** 10.1186/s12890-017-0363-0

**Published:** 2017-02-01

**Authors:** Eef D. Telenga, Matthijs Oudkerk, Peter M. A. van Ooijen, Rozemarijn Vliegenthart, Nick H. T. ten Hacken, Dirkje S. Postma, Maarten van den Berge

**Affiliations:** 1 Department of Pulmonary Diseases, University of Groningen, University Medical Center Groningen, PO Box 30.001, 9700 RB Groningen, The Netherlands; 2 GRIAC Research Institute, University of Groningen, University Medical Center Groningen, Groningen, The Netherlands; 3 Center for Medical Imaging North East Netherlands, University of Groningen, University Medical Center Groningen, Groningen, The Netherlands; 4 Department of Radiology, University of Groningen, University Medical Center Groningen, PO Box 30.001, 9700 RB Groningen, The Netherlands

**Keywords:** Airway wall thickness, Computed tomography, Smoking, Age

## Abstract

**Background:**

To investigate if age, gender and smoking are associated with airway wall thickness (AWT) measured by high resolution computed tomography (HRCT) and if higher AWT is associated with lower levels of pulmonary function in healthy current- and never-smokers with a wide age range.

**Methods:**

HRCT scans were performed in 99 subjects (48 never- and 51 current-smokers, median age 39 years [IQR 22 – 54], 57% males). The AWT at an internal perimeter of 10 mm (AWT Pi10) was calculated as an overall measurement of AWT, based on all measurements throughout the lungs. Extensive pulmonary function testing was performed in all subjects.

**Results:**

Higher age was associated with a lower AWT Pi10 (b = −0.003, *p* < 0.001). Current-smokers had a higher AWT Pi10 than never-smokers (mean 0.49 mm versus 0.44 mm, *p* = 0.022). In multivariate analysis, age and current-smoking were independently associated with AWT Pi10 (age b = −0.002, *p* < 0.001, current-smoking b = 0.041, *p* = 0.021), whereas gender was not (b = 0.011, *p* = 0.552). Higher AWT Pi10 was associated with a lower FEV_1_, FEV_1_/FVC, FEF_25–75_ and higher R5, R20 and X5.

**Conclusions:**

AWT decreases with higher age, possibly reflecting structural changes of the airways. Additionally, current-smokers have a higher AWT, possibly due to remodeling or inflammation. Finally, higher AWT is associated with a lower level of pulmonary function, even in this population of healthy subjects.

**Trial registration:**

This Study was registered at www.clinicaltrials.gov with number NCT00848406 on 19 February 2009.

**Electronic supplementary material:**

The online version of this article (doi:10.1186/s12890-017-0363-0) contains supplementary material, which is available to authorized users.

## Background

Chronic inflammatory processes in the airways, like those occurring in asthma and chronic obstructive pulmonary disease (COPD), induce airway remodeling. This is associated with changes in the epithelial layer, reticular basement membrane and smooth muscle, all contributing to thickening of the airway walls [1, 2].

Computed tomography (CT) can be used as a non-invasive tool to assess airway wall thickness (AWT). Using this technique, it has been shown that the airways walls of patients with COPD are significantly thicker than those of healthy non-smoking controls [3]. In addition, it has been shown that higher AWT is associated with more severe airflow obstruction in COPD [3–10]. Although these studies provided important insights, there were also some drawbacks. Most studies performed thus far used transverse CT slices and were therefore only able to measure airways oriented perpendicular to this plane [4–6, 8, 9, 11]. This is an important limitation as it markedly limits the number of airways that can be measured. Further, some studies measured AWT manually [5, 9, 11], which is less accurate than automated measurements and more susceptible to interobserver variation [12]. Advances in multi-detector CT scanners, development of multiplanar, three-dimensional segmentation of airways and automated measurements of airway walls now make it possible to measure AWT in multiple airways throughout the bronchial tree.

Finally, most previous studies included selective populations of older subjects and often only current- or ex-smokers [13–15]. For this reason, the effects of age and smoking status on AWT are largely unknown. This may be important, since both age and smoking status are likely to affect AWT. Aging has been shown to play a role in remodeling and repair processes in lung parenchyma and similar processes are likely to occur in the airway walls as well [16, 17]. Additionally, smoking is the main risk factor for the development of COPD. It causes airway inflammation and remodeling, both influencing AWT [18–20].

In the present study, we investigated AWT in a group of well characterized healthy subjects. An automated software program was used, measuring a large number of airways perpendicular to the airway direction throughout the lungs. The objectives of this study were to investigate if age, gender and smoking are associated with AWT and whether a higher AWT is associated with lower levels of pulmonary function in healthy current- and never-smokers with a wide age range.

## Methods

### Study population

In this study, healthy never- and current-smokers were included if they met the following criteria: normal spirometry, no bronchial hyperresponsiveness to methacholine and normal pulmonary health according to the physician. Spirometry was considered to be normal if the forced expiratory volume in 1 s (FEV_1_) was ≥80%predicted, the FEV_1_/forced vital capacity (FVC) was greater than the lower limit of normal and reversibility to salbutamol was <10% of the predicted value. Never-smokers were defined as subjects who had not smoked during the last year, had never smoked for as long as 1 year, and had not smoked more than 0.5 packyears. The study was approved by the local medical ethics committee (METc 2007/007) and all subjects gave their written informed consent.

### High Resolution CT scans and airway measurements

High resolution CT (HRCT) scans were performed using a 64-multidetector CT scanner (Somatom Definition, Siemens, Forchheim, Germany). Scans were performed at full inspiration. Scanning was performed with 20 mAs. The kV setting was determined by weight: 100 kV for subjects <60 kg, 120 kV for subjects ≥60 and <80 kg and 140 kV for subjects >80 kg. Acquired imaging data were reconstructed using a standard soft kernel (B30f), with 1.0 mm slice thickness and 0.7 mm increment. The CT radiation dose was approximately 0.8 mSv (100 kV) to 1.5 mSv (140 kV), the (median CT radiation dose was 0.95 mSv.

**Fig. 1:**
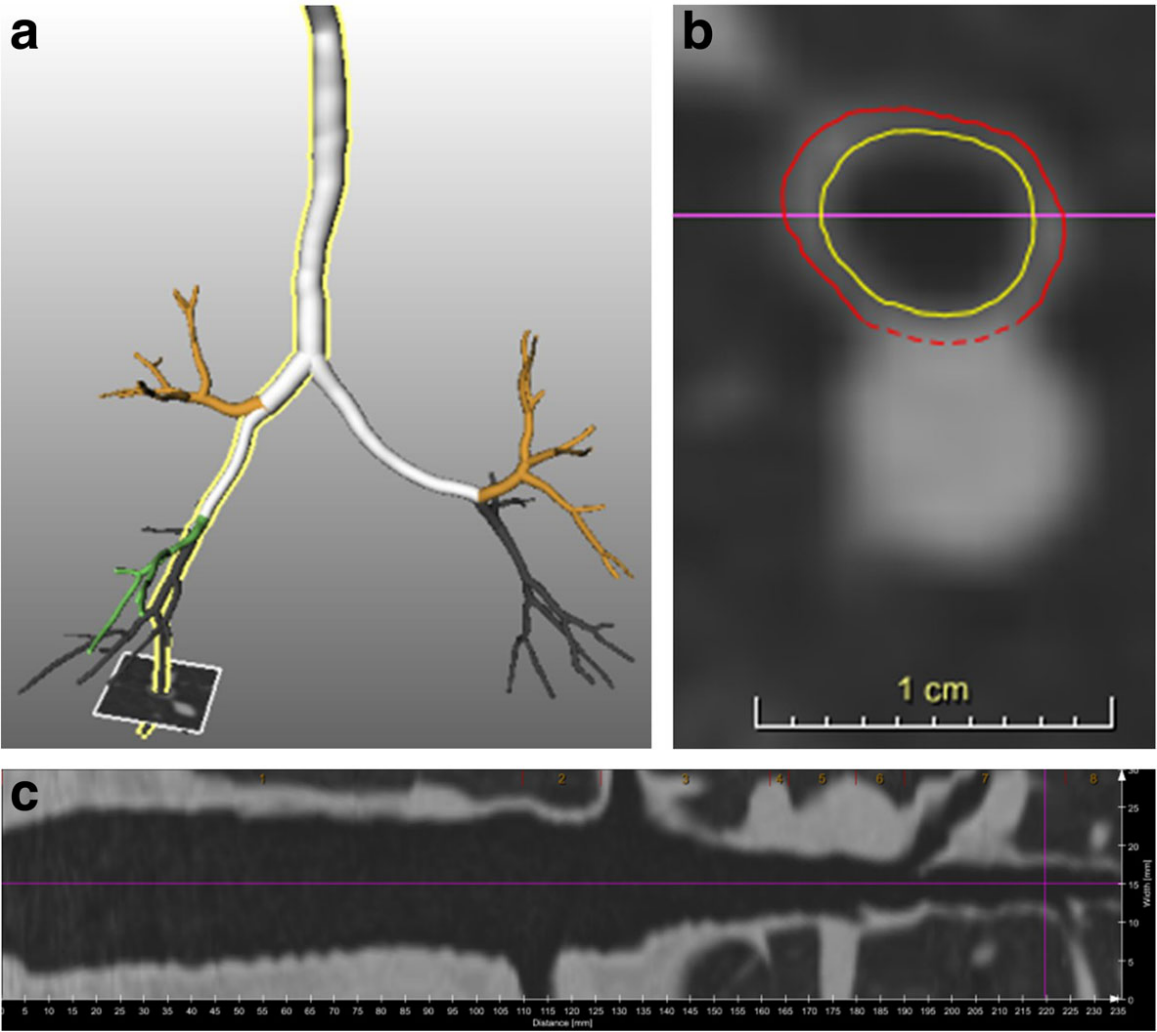
Airway wall thickness analysis. **a** The airway wall tree as extracted by the software program. The airway highlighted in yellow is stretched to a 2D picture in (**b**). The grey square corresponds to the purple vertical line in (**b**). This is a cross sectional image of the airway oriented perpendicular to the local centerline direction and given in more detail in (**c**). The red line is the outer airway wall perimeter and the yellow line is the inner airway wall perimeter. Airway wall thickness (AWT) and wall area percentage (%AWA) are measured in areas of the continuous red line. Dashed parts of the outer perimeter are interpolated and not used for measurements. The assessed perimeter fraction (APF) is the fraction of the outer perimeter that has a continuous line

Airway measurements were performed with the automated software program MeVis Airway Examiner 1.0 (Fraunhofer Institute for Medical Image Computing MEVIS, Bremen, Germany). The software automatically extracts airway centerlines, re-samples images perpendicular to the airway direction and detects inner and outer airway wall borders in these images (Fig. 1) [21]. The outer wall border is detectable when no adjacent tissue with similar CT density is present. All HRCT scans were visually evaluated for appropriate segmentation and data from lobes that were incorrectly segmented were removed from further analyses. The software calculated AWT, airway wall area percentage (%AWA), i.e. airway wall area/total airway area *100, and the fraction of the airway that was detectable (assessed perimeter fraction, APF) for each location and reported the average AWT and %AWA per lung lobe for airways with predefined external diameters (3.5 mm, 4 mm, 4.5 mm, 5 mm, 6 mm, 8 mm and 10 mm, all ±0.25 mm) from all the separately measured airway locations. The software also reported the cumulative APF per lung lobe for each external diameter measured, i.e. the total number of airway perimeters measured, calculated by adding up the APF of all measured airways with the appropriate external diameter. A higher cumulative APF reflects a higher number of sampling points assessed. The mean AWT and %AWA per lung for each external diameter were calculated as the weighted average of the AWT and %AWA per lobe, with the cumulative APF per lobe as the weighting factor. As an overall measurement of AWT, the AWT at an internal perimeter of 10 mm (AWT Pi10) was estimated based on all measurements at the 7 different diameters, similar to the method of Grydeland et al. [13]. The AWT Pi10 was calculated by plotting the square root of the average area of airway wall per lobe against the internal perimeter of these measurements. The resulting regression line was used to calculate the AWT Pi10. Our method differed slightly from the Grydeland et al. in that they used all measured airways, instead of airways with predefined diameters [13].

### Pulmonary function tests

Spirometry was performed before and after 400 μg salbutamol according to current ATS/ERS guidelines [22, 23]. Impulse oscillometry (IOS) was performed to measure resistance at 5 Hz (R5), resistance at 20 Hz (R20), the difference between R5 and R20 (R5-20) and reactance at 5 Hz (X5). Body plethysmography was performed to measure functional residual capacity (FRC), total lung capacity (TLC) and residual volume (RV). The diffusion capacity of the lung for carbon monoxide corrected for hemoglobin level (TLCOc) and TLCOc corrected for the alveolar volume (TLCOc/VA) were measured. Provocation tests were performed with methacholine (0.03 to 16 mg/ml), using a 2-min tidal breathing method, adapted from Cockcroft et al. [24].

### Statistical analysis

Differences in AWT Pi10, AWT and %AWA were tested with Student’s *t*-test or analysis of variance (ANOVA), with post-hoc Holm’s Bonferroni correction for multiple testing. To assess the effect of age on airway wall thickness, linear regressions were performed with either AWT Pi10, AWT or %AWA as the outcome variable and age, gender and smoking status as predictor variables. To assess the effect of airway wall thickness on pulmonary function parameters, multivariate regression analyses with the pulmonary function parameter as outcome variable and AWT Pi10 as predictor variable with adjustment for age, gender and smoking status were performed. If height is part of the formula to calculate the predicted value of a pulmonary function parameter, height was also adjusted for. Similar analyses were performed with the AWT and %AWA transformed to the number of standard deviations (SD) from the mean at the different diameters. All statistical analyses were performed with IBM SPSS version 20 (IBM, Armonk, NY, USA). *P*-values <0.05 were considered statistically significant.

## Results

A total of 99 subjects were included. Their baseline characteristics are presented in Table 1. In 25 patients the software was not able to detect the airways correctly in the entire lung and the data from lobes that were incorrectly segmented were removed from further analyses. Data from one lobe was removed from the analysis for 21 patients and data from two lobes was removed in 4 patients. The median cumulative assessed perimeter fractions (APF) per patient and per lung lobe are presented in Additional file 1: Table S1. The median cumulative APF per patient was 146 airways, reflecting that the AWT was measured on a total 146 airway perimeters. The cumulative APF was highest for airways with 5 mm external diameter. At this diameter the software measured a median of 55 airway perimeters. The software was able to measure wall thickness of all subjects between 4.5 and 6 mm. At smaller diameters there were less successful measurements (96 of 99 subjects at 4 mm and 58 of 99 subjects at 3.5 mm). Furthermore the number of airways measured became very limited at 3.5 mm with a cumulative APF of only 0.77 airways. Below 3.5 mm no airways were detected by the software. At higher diameters the APF also decreased to 1.31 airways at 10 mm. The lowest cumulative APF was observed in the right middle lobe, reflecting the smaller size of this lobe.

**Table 1:** Baseline characteristics of the 99 subjects

Age (years)	39	(22 – 54)
Males (n, %)	56	(57)
Current-smokers (n, %)	51	(52)
Cigarettes/day (n)^a^	15	(3 – 29)
Packyears (n)^a^	15	(10 – 20)
FEV_1_ (%predicted)	105	(98 – 113)
FVC (%predicted)	113	(105 – 118)
FEV_1_/FVC (%)	79	(76 – 84)
FEF_25–75_ (%predicted)	83	(71 – 99)
RV/TLC (%predicted)	86	(77 – 96)
TLCOc/VA (%predicted)	96	(88 – 104)
R5 (kPa/L/s)	0.30	(0.25 – 0.36)
R20 (kPa/L/s)	0.28	(0.22 – 0.33)
R5-20 (kPa/L/s)	0.02	(0.00 – 0.05)
X5 (kPa/L/s)	−0.07	(−0.10 – −0.06)

Values are presented as medians with interquartile ranges unless stated otherwise

*FEV*_*1*_ forced expiratory volume in 1 s, *FVC* forced vital capacity, *FEF*_*25–75*_ forced expiratory flow between 25 and 75% of FVC, *RV/TLC* residual volume/total lung capacity, *TLCOc* diffusion capacity of the lung for carbon monoxide, corrected for hemoglobin level, *VA* alveolar volume, *R5* resistance at 5 Hz, *R20* resistance at 20Hz, *R5-20* difference between R5 and R20, *X5* reactance at 5 Hz, *AX* reactance area

^a^only in current-smokers

### AWT and %AWA at different diameters and in different lobes

AWT increased linearly with increasing diameter from 0.30 mm to 3.5 mm external diameter to 1.42 mm at 10 mm (Additional file 2: Figure S1a). The %AWA was 29.8% at 3.5 mm external diameter and increased to 45.1% at 6 mm after this the %AWA reached a plateau around 48% (Additional file 2: Figure S1b). The AWT and %AWA were comparable in all lobes for external diameters between 4 and 10 mm (Additional file 2: Figure S1c and d and Additional file 1: Tables S2 and S3). At 3.5 mm the AWT and %AWA were significantly higher in the left lower lobe than in the right upper lobe.

### Effect of age, gender and smoking status on AWT Pi10, AWT and %AWA

**Fig. 2:**
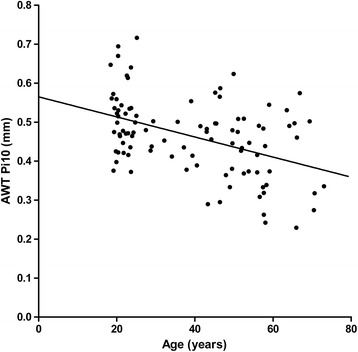
Association between AWT Pi10 and age. b = −0.003, *p* < 0.001; AWT Pi10 = airway wall thickness at an internal perimeter of 10 mm estimated from all measurements

**Fig. 3:**
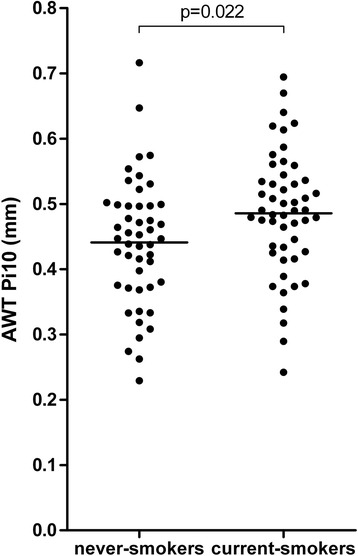
AWT Pi10 in never- and current-smokers. AWT Pi10 = airway wall thickness at an internal perimeter of 10 mm estimated from all measurements

Higher age was associated with lower AWT, assessed by the overall measure of airway wall thickness, the AWT Pi10 (b = −0.003, *p* < 0.001, Fig. 2). A trend was found towards a lower AWT Pi10 in men compared to women (mean (SD) 0.45 (0.10) mm versus 0.48 (0.09) mm respectively, *p* = 0.088). AWT Pi10 was significantly higher in current- than in never-smokers (mean (SD) 0.49 (0.09) mm and 0.44 (0.10) mm respectively, *p* = 0.022, Fig. 3). AWT and %AWT were also higher in current- than never-smokers at 4 mm, 4.5 mm, 5 mm, 6 mm and 8 mm diameter, but not at 3.5 mm and 10 mm (Additional file 1: Tables S4 and S5). In multivariate linear regression analysis, both age and smoking status were significantly and independently associated with the AWT Pi10. AWT Pi10 was lower at higher age (b = −0.002, *p* < 0.001) and higher in current-smokers (b = 0.041, *p* = 0.021). Gender was not independently associated with AWT Pi10 (b = 0.011, *p* = 0.552). No significant interaction between age and smoking status was found (*p* = 0.45). In similar analyses, age was negatively associated with AWT and %AWA for all pre-defined diameters, except for 10 mm and current-smoking was associated with a higher AWT and %AWA at 4.5 mm, 5 mm and 6 mm diameter (Additional file 1: Tables S6 and S7).

### Association between airway wall thickness and pulmonary function

A higher AWT Pi10 was associated with lower FEV_1_, FEF_25–75_, FVC, FEV_1_/FVC and X5 and higher R5 and R20, independently from age, gender, height and smoking status (Table 2). AWT Pi10 was not associated with RV/TLC or R5-20. Additionally, a higher AWT Pi10 was associated with higher TLCOc/VA. Similar associations were seen for AWT and %AWA at the different airway diameters (Additional file 1: Tables S8 and S9). The regression coefficients, representing the change in a pulmonary function test for every standard deviation change in AWT or %AWA, were very similar for the different diameters.

**Table 2:** Association between AWT Pi10 and pulmonary function parameters

	AWT Pi10	
	b	*p*-value
FEV_1_ (L)	−1.697	0.001
FEF_25–75_ (L/s)	−3.215	0.001
FVC (L)	−1.288	0.047
FEV_1_/FVC (%)	−13.358	0.018
RV/TLC (%)	3.202	0.468
TLCOc/VA (mmol/min/kPa/L)	0.431	0.029
R5 (kPa/L/s)	0.363	<0.001
R20 (kPa/L/s)	0.288	<0.001
R5-20 (kPa/L/s)	0.075	0.164
X5 (kPa/L/s)	−0.080	0.028

Linear regressions with pulmonary function parameters as outcome and airway wall thickness as predictor variable and age, gender, smoking status and height added as covariates

*AWT Pi10* airway wall thickness at an internal perimeter of 10 mm estimated from all measurements, *FEV*_*1*_ forced expiratory volume in 1 s, *FVC* forced vital capacity, *FEF*_*25–75*_ forced expiratory flow between 25 and 75% of FVC, *RV/TLC* residual volume/total lung capacity, *TLCOc* diffusion capacity of the lung for carbon monoxide, corrected for hemoglobin level, *VA* alveolar volume, *R5* resistance at 5 Hz, *R20* resistance at 20Hz, *R5-20* difference between R5 and R20, *X5* reactance at 5 Hz

## Discussion

The present study demonstrates that airway wall thickness (AWT) decreases with aging and is higher in current- than never-smokers. In addition, we show that thicker airway walls are associated with lower levels of pulmonary function.

The effect of age on AWT has been previously described by Grydeland et al. in COPD patients and healthy smokers >40 years [13]. In this study, the estimated AWT at an internal perimeter of 10 mm (AWT Pi10) decreased with 0.003 mm per year in the healthy smokers, which is very similar to the results of this study (0.002 mm per year in the multivariate analysis). These findings are partly in agreement with a study by Zach et al. [14]. In this study, age did not contribute to AWT Pi10 in 92 healthy subjects >45 years. However, a higher age was associated with a lower %AWA. We extend the data of Zach et al. by also including younger subjects and never-smokers. This way, we were able to show that airway walls become thinner from early adulthood on throughout life and that this process is independent from smoking. On the other hand, in a study by Matsuoka et al. no differences were found in the ratio of airway wall thickness and the total diameter of the bronchus in healthy subjects aged 21–40, 41–65 and >65 years old [11]. However in this study, only transverse CT slices were used, thereby limiting the number of airways measured. Furthermore, the airways were measured manually, which is less accurate than automated measurements and more susceptible to interobserver variation [12].

Aging has profound influences on the lung. The elastic recoil of the lung decreases significantly with aging, mainly due to alveolar enlargement without alveolar wall destruction, a condition that is also called ‘senile lung’ [16]. It has been suggested that remodeling of the extracellular matrix (ECM) in the lung parenchyma, with degradation of elastin and collagens, is the main factor in this aging process [16, 17]. However, there is limited knowledge of the effects of aging on changes in the airway walls. Animal studies suggest that aging of Clara cells, the progenitor cells of the airway epithelium, may impair airway regeneration, which could lead to thinning of the airway walls [25]. It is also possible that, similar to the parenchyma, ECM proteins are degraded in the airway walls with aging. ECM proteins also degrade with age in many other structures in the human body, for instance the skin [26]. Interestingly, in the aging skin, an altered function of fibroblasts has been observed, resulting in a decreased production of collagen and increased production of collagen-degrading matrix metalloproteinases. It is tempting to speculate that comparable changes may occur in aging airway fibroblasts. Alternatively, inflammatory cells and other residential cells, like epithelial and smooth muscle cells, may have similar effects.

This study demonstrates that current-smokers have a higher AWT than never-smokers, independently from age. Donohue et al. did not find higher thickness of airway walls in current-smokers than in never- or ex-smokers in a large cohort of ex-, current- and never-smokers [15]. However, they did show that airway walls were thicker with more cigarette exposure, i.e. more packyears smoked and that current-smoking was associated with a narrower airway lumen. In the study by Matsuoka et al. the in the ratio of airway wall thickness and the total diameter of the bronchus was higher in smokers aged >65 than never-smokers aged >65. No differences were seen between smokers and never-smokers <65 years [11]. Thicker airway walls in current-smokers may be due to the effects of continuous smoke exposure on the epithelium, causing the epithelium to produce pro-inflammatory cytokines that induce remodeling [18–20]. In line with this, a study in 25 smokers and 14 non-smokers showed more goblet cell metaplasia, more smooth muscle hypertrophy and more inflammation in the walls of airways in current-smokers [27]. Thus, smoking may affect AWT in several different ways. It is not possible to distinguish between the different causes of airway wall thickening on HRCT scans.

An increase in AWT may be of clinical relevance, since it is associated with lower levels of pulmonary function, independent of age, gender, height and smoking status. Correlations between pulmonary function and wall thickness have previously been demonstrated in studies with COPD patients and older healthy controls [3, 5–9]. In this study, associations between AWT and pulmonary function are demonstrated in a population of subjects who were specifically selected to be asymptomatic and have normal spirometry. Interestingly, AWT was associated not only with large airway parameters like the FEV_1_, FVC and FEV_1_/FVC, but also with small airway parameters like the FEF_25–75_ and X5. This is remarkable since the small airways have an internal diameter <2 mm and are therefore too small to be measured on HRCT scans at the moment. Additionally, an increase of 1 SD in AWT in airways with smaller diameters (e.g. 4 or 4.5 mm) or larger diameters (e.g. 6 or 8 mm) had similar effects on pulmonary function tests measuring large and small airways (Additional file 1: Tables S8 and S9). These results suggest that thickening of airway walls in current-smokers occurs not only in the airways that were measured, but throughout the lungs. Advances in ultra-high resolution imaging, like cone beam CT imaging, may provide more information about the AWT in the small airways in the future [28, 29]. Unexpectedly, thicker airway walls were associated with a higher TLCOc/VA. As it is unlikely that subjects with thicker airway walls truly have a higher diffusion capacity, we hypothesize that the increased diffusion capacity in subjects with thicker airway walls most likely results from underestimation of the alveolar volume due to ventilation inhomogeneity [30].

There are several strengths to our study. First, we used a software program automatically detecting the bronchial tree and measuring AWT perpendicular to the airway direction. Using this method, we were able to measure a median of 146 airway perimeters (combined from partial airway perimeters) per patient. These measurements were performed at different airway diameters, ranging from 3.5 to 10 mm external diameter, in all lung lobes. Second, all HRCT scans were performed in the same center, using the same acquisition device settings. Finally, all subjects were extensively characterized with pulmonary function tests, smoking and medical history. There are also limitations to this study. As AWT was measured at pre-defined external airway parameters, it is not possible to determine if increases in AWT are due to actual thickening of the wall or due to reduction in the luminal diameter. This is a general problem with measuring AWT. A possible solution could be to assess airways per generation. However, currently, it is still difficult to do this and labeling of airway generation is prone to error.

## Conclusions

In conclusion, airway wall thickness measured with HRCT scans is a feasible technique to investigate anatomical changes of the airways. We showed that airway wall thickness decreases with age, possibly reflecting structural changes of the airways during aging. Additionally, current-smokers have thicker airway walls than never-smokers, and this increased airway wall thickness is associated with lower levels of pulmonary function.

## Additional files

Additional file 1: Table S1. Cumulative APF at the different airway diameters per lung lobe. **Table S2.** Airway wall thickness per lobe. **Table S3.** Airway wall area per lobe. **Table S4.** Airway wall thickness according to smoking status. **Table S5.** Airway wall area according to smoking status. **Table S6:** Associations between airway wall thickness and age, gender and smoking status. **Table S7.** Associations between airway wall area and age, gender and smoking status. **Table S8.** Association between pulmonary function parameters and airway wall thickness, independent of age, gender, smoking status and height. **Table S9.** Association between pulmonary function parameters and airway wall area, independent of age, gender, smoking status and height. (DOCX 51 kb)
Additional file 2: Figure S1. AWT and %AWA at different external airway diameters. AWT = airway wall thickness; %AWA = airway wall area percentage. (TIF 3044 kb)

## Abbreviations

%AWA: Airway wall area percentage
APF: Assessed perimeter fraction
AWT: Airway wall thickness
AWT: Pi10 airway wall thickness at an internal perimeter of 10 mm
COPD: Chronic obstructive pulmonary disease
CT: Computed tomography
ECM: Extracellular matrix
FEF_25–75_: Forced expiratory flow between 25 and 75% of FVC
FEV_1_: Forced expiratory volume in 1 s
FRC: Functional residual capacity
FVC: Forced vital capacity
HRCT: High resolution computed tomography
IOS: Impulse oscillometry
R20: Resistance at 20 Hz
R5: Resistance at 5 Hz
R5-20: Difference between R5 and R20
RV: Residual volume
TLC: Total lung capacity
TLCOc: Diffusion capacity of the lung for carbon monoxide corrected for hemoglobin level
TLCOc/VA: TLCOc corrected for the alveolar volume
X5: Reactance at 5 Hz

## References

[1] Nagai A, West WW, Thurlbeck WM (1985). The National Institutes of Health Intermittent Positive-Pressure Breathing trial: pathology studies. II. Correlation between morphologic findings, clinical findings, and evidence of expiratory air-flow obstruction. Am Rev Respir Dis.

[2] Bosken CH, Wiggs BR, Pare PD, Hogg JC (1990). Small airway dimensions in smokers with obstruction to airflow. Am Rev Respir Dis.

[3] Kurashima K, Hoshi T, Takayanagi N, Takaku Y, Kagiyama N, Ohta C, Fujimura M, Sugita Y (2012). Airway dimensions and pulmonary function in chronic obstructive pulmonary disease and bronchial asthma. Respirology.

[4] Berger P, Perot V, Desbarats P, Tunon-de-Lara JM, Marthan R, Laurent F (2005). Airway wall thickness in cigarette smokers: quantitative thin-section CT assessment. Radiology.

[5] Arakawa H, Fujimoto K, Fukushima Y, Kaji Y (2011). Thin-section CT imaging that correlates with pulmonary function tests in obstructive airway disease. Eur J Radiol.

[6] Nakano Y, Muro S, Sakai H, Hirai T, Chin K, Tsukino M, Nishimura K, Itoh H, Pare PD, Hogg JC, Mishima M (2000). Computed tomographic measurements of airway dimensions and emphysema in smokers. Correlation with lung function. Am J Respir Crit Care Med.

[7] Hasegawa M, Nasuhara Y, Onodera Y, Makita H, Nagai K, Fuke S, Ito Y, Betsuyaku T, Nishimura M (2006). Airflow limitation and airway dimensions in chronic obstructive pulmonary disease. Am J Respir Crit Care Med.

[8] Patel BD, Coxson HO, Pillai SG, Agusti AG, Calverley PM, Donner CF, Make BJ, Muller NL, Rennard SI, Vestbo J, Wouters EF, Hiorns MP, Nakano Y, Camp PG, Nasute Fauerbach PV, Screaton NJ, Campbell EJ, Anderson WH, Pare PD, Levy RD, Lake SL, Silverman EK, Lomas DA, International COPD Genetics Network (2008). Airway wall thickening and emphysema show independent familial aggregation in chronic obstructive pulmonary disease. Am J Respir Crit Care Med.

[9] Orlandi I, Moroni C, Camiciottoli G, Bartolucci M, Pistolesi M, Villari N, Mascalchi M (2005). Chronic obstructive pulmonary disease: thin-section CT measurement of airway wall thickness and lung attenuation. Radiology.

[10] Xie X, de Jong PA, Oudkerk M, Wang Y, Ten Hacken NH, Miao J, Zhang G, de Bock GH, Vliegenthart R (2012). Morphological measurements in computed tomography correlate with airflow obstruction in chronic obstructive pulmonary disease: systematic review and meta-analysis. Eur Radiol.

[11] Matsuoka S, Uchiyama K, Shima H, Ueno N, Oish S, Nojiri Y (2003). Bronchoarterial ratio and bronchial wall thickness on high-resolution CT in asymptomatic subjects: correlation with age and smoking. AJR Am J Roentgenol.

[12] Reinhardt JM, D’Souza ND, Hoffman EA (1997). Accurate measurement of intrathoracic airways. IEEE Trans Med Imaging.

[13] Grydeland TB, Dirksen A, Coxson HO, Pillai SG, Sharma S, Eide GE, Gulsvik A, Bakke PS (2009). Quantitative computed tomography: emphysema and airway wall thickness by sex, age and smoking. Eur Respir J.

[14] Zach JA, Newell JD, Schroeder J, Murphy JR, Curran-Everett D, Hoffman EA, Westgate PM, Han MK, Silverman EK, Crapo JD, Lynch DA, on behalf of the COPDGene Investigators (2012). Quantitative Computed Tomography of the Lungs and Airways in Healthy Nonsmoking Adults. Invest Radiol.

[15] Donohue KM, Hoffman EA, Baumhauer H, Guo J, Budoff M, Austin JH, Kalhan R, Kawut S, Tracy R, Graham Barr R (2012). Cigarette smoking and airway wall thickness on CT scan in a multi-ethnic cohort: The MESA Lung Study. Respir Med.

[16] Fukuchi Y (2009). The aging lung and chronic obstructive pulmonary disease: similarity and difference. Proc Am Thorac Soc.

[17] Knudson RJ, Clark DF, Kennedy TC, Knudson DE (1977). Effect of aging alone on mechanical properties of the normal adult human lung. J Appl Physiol.

[18] Takizawa H, Tanaka M, Takami K, Ohtoshi T, Ito K, Satoh M, Okada Y, Yamasawa F, Nakahara K, Umeda A (2001). Increased expression of transforming growth factor-beta1 in small airway epithelium from tobacco smokers and patients with chronic obstructive pulmonary disease (COPD). Am J Respir Crit Care Med.

[19] Vignola AM, Chanez P, Chiappara G, Merendino A, Pace E, Rizzo A, la Rocca AM, Bellia V, Bonsignore G, Bousquet J (1997). Transforming growth factor-beta expression in mucosal biopsies in asthma and chronic bronchitis. Am J Respir Crit Care Med.

[20] Morris DG, Huang X, Kaminski N, Wang Y, Shapiro SD, Dolganov G, Glick A, Sheppard D (2003). Loss of integrin alpha(v)beta6-mediated TGF-beta activation causes Mmp12-dependent emphysema. Nature.

[21] Weinheimer O, Achenbach T, Bletz C, Duber C, Kauczor HU, Heussel CP (2008). About objective 3-d analysis of airway geometry in computerized tomography. IEEE Trans Med Imaging.

[22] Miller MR, Hankinson J, Brusasco V, Burgos F, Casaburi R, Coates A, Crapo R, Enright P, van der Grinten CP, Gustafsson P, Jensen R, Johnson DC, MacIntyre N, McKay R, Navajas D, Pedersen OF, Pellegrino R, Viegi G, Wanger J, ATS/ERS Task Force (2005). Standardisation of spirometry. Eur Respir J.

[23] Quanjer PH, Tammeling GJ, Cotes JE, Pedersen OF, Peslin R, Yernault JC (1994). Lung volumes and forced ventilatory flows. Work Group on Standardization of Respiratory Function Tests. European Community for Coal and Steel. Official position of the European Respiratory Society. Rev Mal Respir.

[24] Cockcroft DW, Killian DN, Mellon JJ, Hargreave FE (1977). Bronchial reactivity to inhaled histamine: a method and clinical survey. Clin Allergy.

[25] Aoshiba K, Nagai A (2009). Senescence hypothesis for the pathogenetic mechanism of chronic obstructive pulmonary disease. Proc Am Thorac Soc.

[26] Naylor EC, Watson RE, Sherratt MJ (2011). Molecular aspects of skin ageing. Maturitas.

[27] Cosio MG, Hale KA, Niewoehner DE (1980). Morphologic and morphometric effects of prolonged cigarette smoking on the small airways. Am Rev Respir Dis.

[28] King GG (2012). Tomographic imaging of small airways. Respiration.

[29] Miracle AC, Mukherji SK (2009). Conebeam CT of the head and neck, part 1: physical principles. Am J Neuroradiol.

[30] Cotton DJ, Mink JT, Graham BL (1998). Nonuniformity of diffusing capacity from small alveolar gas samples is increased in smokers. Can Respir J.

