# Supplementary material for: Airway wall thickness on HRCT scans decreases with age and increases with smoking
Source: BMC Pulm Med. 2017 Feb 1;17:27. doi: 10.1186/s12890-017-0363-0 (PMC5286807; doi:10.1186/s12890-017-0363-0)
Supplement: Additional file 1: Table S1. — Cumulative APF at the different airway diameters per lung lobe. Table S2. Airway wall thickness per lobe. Table S3. Airway wall area per lobe. Table S4. Airway wall thickness according to smoking status. Table S5. Airway wall area according to smoking status. Table S6: Associations between airway wall thickness and age, gender and smoking status. Table S7. Associations between airway wall area and age, gender and smoking status. Table S8. Association between pulmonary function parameters and airway wall thickness, independent of age, gender, smoking status and height. Table S9. Association between pulmonary function parameters and airway wall area, independent of age, gender, smoking status and height. (DOCX 51 kb) [file 12890_2017_363_MOESM1_ESM.docx]

**Table S1:** Cumulative APF at the different airway diameters per lung lobe

| External perimeter | **Total** | | | **RUL** | | | **RML** | | | **RLL** | | | | | **LUL** | | | | | **LLL** | | |
| --- | --- | --- | --- | --- | --- | --- | --- | --- | --- | --- | --- | --- | --- | --- | --- | --- | --- | --- | --- | --- | --- | --- |
|  | **Median / IQR** | | **n=** | **Median / IQR** | | **n=** | **Median / IQR** | | **n=** | **Median / IQR** | | | **n=** | | **Median / IQR** | | | **n=** | | **Median / IQR** | | **n=** |
| 3.5 mm | 0.8 | (0.3 – 1.5) | 58 | 0.7 | (0.3 – 1.5) | 20 | 0.5 | (0.1 – 0.8) | 15 | | 0.4 | (0.2 – 1.0) | | 33 | | 0.6 | (0.1 – 0.8) | 18 | 0.3 | | (0.1 – 0.7) | 27 |
| 4 mm | 9.4 | (4.3 – 17.4) | 96 | 2.9 | (1.0 – 5.3) | 66 | 1.2 | (0.7 – 2.5) | 61 | | 3.4 | (1.5 – 6.5) | | 77 | | 2.3 | (1.0 – 4.3) | 79 | 1.8 | | (1.0 – 3.7) | 69 |
| 4.5 mm | 41.0 | (25.8 – 65.7) | 99 | 9.1 | (4.9 – 14.0) | 95 | 4.0 | (2.1 – 6.1) | 84 | | 13.1 | (7.3 – 20.6) | | 91 | | 10.4 | (4.9 – 16.2) | 91 | 9.4 | | (3.8 – 14.3) | 88 |
| 5 mm | 55.3 | (39.9 – 74.1) | 99 | 11.2 | 7.1 – 17.1) | 97 | 4.2 | (2.3 – 7.5) | 95 | | 15.7 | (11.5 – 22.2) | | 92 | | 14.4 | (7.4 – 19.7) | 93 | 13.2 | | (7.1 – 19.0) | 88 |
| 6 mm | 32.6 | (22.8 – 46.8) | 99 | 5.9 | (3.2 – 9.8) | 96 | 3.2 | (1.8 – 4.9) | 93 | | 9.6 | (5.6 – 15.0) | | 92 | | 6.7 | (4.1 – 9.7) | 90 | 7.3 | | (4.0 – 12.6) | 87 |
| 8 mm | 5.3 | (3.0 – 9.8) | 98 | 0.9 | (0.3 – 1.7) | 69 | 0.6 | (0.2 – 1.2) | 75 | | 1.9 | (0.7 – 3.8) | | 81 | | 1.0 | (0.6 – 1.7) | 80 | 1.7 | | (1.0 – 3.3) | 85 |
| 10 mm | 1.3 | (0.6 – 2.7) | 94 | 0.3 | (0.2 – 0.6) | 45 | 0.3 | (0.1 – 0.5) | 55 | | 0.6 | (0.2 – 1.3) | | 62 | | 0.6 | (0.2 – 1.5) | 82 | 0.3 | | (0.2 – 0.7) | 54 |
| Total | 146.1 | (110.5 – 207.6) | 99 | 33.1 | (22.4 – 44.1) | 97 | 13.3 | (8.0 – 19.8) | 99 | | 44.8 | (31.3 – 63.4) | | 96 | | 33.3 | (21.2 – 51.2) | 98 | 30.8 | | (17.0 – 46.1) | 99 |

APF = assessed perimeter fraction; RUL = right upper lobe; RML = right middle lobe; RLL = right lower lobe; LUL = left upper lobe; LLL = left lower lobe; IQR = interquartile range.

**Table S2:** Airway wall thickness per lobe

|  | **Total** |  |  |  | **RUL** |  |  |  | **RML** |  |  |  | **RLL** |  |  |  | **LUL** |  |  |  | **LLL** |  |  |  | **p-value** |
| --- | --- | --- | --- | --- | --- | --- | --- | --- | --- | --- | --- | --- | --- | --- | --- | --- | --- | --- | --- | --- | --- | --- | --- | --- | --- |
|  | **mean** | **SD** | **n=** |  | **mean** | **SD** | **n=** |  | **mean** | **SD** | **n=** |  | **mean** | **SD** | **n=** |  | **mean** | **SD** | **n=** |  | **mean** | **SD** | **n=** |  |  |
| AWT @3.5 mm | 0.30 | 0.08 | 58 |  | 0.24 | 0.09 | 20 |  | 0.28 | 0.09 | 15 |  | 0.32 | 0.10 | 33 |  | 0.29 | 0.08 | 18 |  | 0.32* | 0.09 | 27 |  | 0.026 |
| AWT @4 mm | 0.40 | 0.10 | 96 |  | 0.40 | 0.11 | 66 |  | 0.39 | 0.10 | 61 |  | 0.40 | 0.09 | 77 |  | 0.40 | 0.09 | 79 |  | 0.40 | 0.11 | 69 |  | 0.965 |
| AWT @4.5 mm | 0.50 | 0.11 | 99 |  | 0.52 | 0.13 | 95 |  | 0.49 | 0.12 | 84 |  | 0.49 | 0.11 | 91 |  | 0.51 | 0.13 | 91 |  | 0.52 | 0.11 | 88 |  | 0.433 |
| AWT @5 mm | 0.60 | 0.13 | 99 |  | 0.62 | 0.14 | 97 |  | 0.62 | 0.14 | 95 |  | 0.60 | 0.13 | 92 |  | 0.62 | 0.15 | 93 |  | 0.62 | 0.13 | 88 |  | 0.888 |
| AWT @6 mm | 0.78 | 0.14 | 99 |  | 0.80 | 0.17 | 96 |  | 0.80 | 0.15 | 93 |  | 0.78 | 0.16 | 92 |  | 0.79 | 0.17 | 90 |  | 0.80 | 0.15 | 97 |  | 0.852 |
| AWT @8 mm | 1.13 | 0.18 | 98 |  | 1.16 | 0.28 | 69 |  | 1.17 | 0.27 | 75 |  | 1.15 | 0.24 | 81 |  | 1.16 | 0.24 | 80 |  | 1.13 | 0.22 | 85 |  | 0.838 |
| AWT @10 mm | 1.42 | 0.21 | 94 |  | 1.38 | 0.32 | 45 |  | 1.50 | 0.31 | 55 |  | 1.35 | 0.32 | 62 |  | 1.40 | 0.28 | 54 |  | 1.37 | 0.33 | 82 |  | 0.070 |

* significantly different from RUL; RUL = right upper lobe; RML = right middle lobe; RLL = right lower lobe; LUL = left upper lobe; LLL = left lower lobe; SD = standard deviation.

**Table S3:** Airway wall area per lobe

|  | **Total** |  |  |  | **RUL** |  |  |  | **RML** |  |  |  | **RLL** |  |  |  | **LUL** |  |  |  | **LLL** |  |  |  | **p-value** |
| --- | --- | --- | --- | --- | --- | --- | --- | --- | --- | --- | --- | --- | --- | --- | --- | --- | --- | --- | --- | --- | --- | --- | --- | --- | --- |
|  | **mean** | **SD** | **n=** |  | **mean** | **SD** | **n=** |  | **mean** | **SD** | **n=** |  | **mean** | **SD** | **n=** |  | **mean** | **SD** | **n=** |  | **mean** | **SD** | **n=** |  |  |
| %AWA @3.5 mm | 29.8 | 7.5 | 58 |  | 24.9 | 8.7 | 20 |  | 26.4 | 10.6 | 15 |  | 31.5 | 8.7 | 33 |  | 29.3 | 7.0 | 18 |  | 32.5* | 8.4 | 27 |  | 0.017 |
| %AWA @4 mm | 34.7 | 7.4 | 96 |  | 34.6 | 8.5 | 66 |  | 34.1 | 8.0 | 61 |  | 35.1 | 7.0 | 77 |  | 34.7 | 7.4 | 79 |  | 34.8 | 8.3 | 69 |  | 0.961 |
| %AWA @4.5 mm | 38.7 | 7.5 | 99 |  | 39.8 | 8.3 | 95 |  | 38.5 | 8.3 | 84 |  | 38.5 | 7.2 | 91 |  | 39.6 | 8.6 | 91 |  | 40.0 | 7.7 | 88 |  | 0.565 |
| %AWA @5 mm | 42.0 | 7.7 | 99 |  | 43.0 | 8.2 | 97 |  | 43.2 | 8.4 | 95 |  | 42.1 | 8.1 | 92 |  | 42.7 | 8.5 | 93 |  | 42.9 | 7.6 | 88 |  | 0.912 |
| %AWA @6 mm | 45.1 | 7.0 | 99 |  | 45.9 | 8.3 | 96 |  | 46.0 | 7.4 | 93 |  | 44.7 | 7.7 | 92 |  | 45.7 | 8.1 | 90 |  | 45.9 | 7.4 | 97 |  | 0.806 |
| %AWA @8 mm | 48.3 | 6.6 | 98 |  | 49.1 | 9.7 | 69 |  | 49.3 | 9.3 | 75 |  | 48.7 | 8.5 | 81 |  | 49.3 | 8.6 | 80 |  | 48.1 | 8.1 | 85 |  | 0.883 |
| %AWA @10 mm | 48.4 | 6.0 | 94 |  | 47.3 | 9.4 | 45 |  | 50.8 | 9.0 | 55 |  | 46.2 | 9.5 | 62 |  | 47.7 | 8.2 | 54 |  | 46.7 | 9.7 | 82 |  | 0.070 |

p-value for ANOVA between the different lobes, followed by post-hoc testing with Holm’s Bonferroni correction; * significantly different from RUL; RUL = right upper lobe; RML = right middle lobe; RLL = right lower lobe; LUL = left upper lobe; LLL = left lower lobe; %AWA = airway wall area percentage; SD = standard deviation;

**Table S4:** Airway wall thickness according to smoking status

|  | **Never-smokers** | | |  | **Current-smokers** | | | **p-value** |
| --- | --- | --- | --- | --- | --- | --- | --- | --- |
|  | mean | SD | n= |  | mean | SD | n= |  |
| AWT @3.5 mm | 0.28 | 0.09 | 33 |  | 0.31 | 0.07 | 25 | 0.219 |
| AWT @4 mm | 0.38 | 0.10 | 46 |  | 0.42 | 0.09 | 50 | 0.048 |
| AWT @4.5 mm | 0.47 | 0.11 | 48 |  | 0.52 | 0.10 | 51 | 0.032 |
| AWT @5 mm | 0.57 | 0.13 | 48 |  | 0.63 | 0.12 | 51 | 0.029 |
| AWT @6 mm | 0.75 | 0.14 | 48 |  | 0.82 | 0.13 | 51 | 0.013 |
| AWT @8 mm | 1.01 | 0.19 | 48 |  | 1.17 | 0.17 | 50 | 0.053 |
| AWT @10 mm | 1.39 | 0.23 | 47 |  | 1.44 | 0.20 | 47 | 0.250 |

AWT = airway wall thickness; SD = standard deviation.

**Table S5:** Airway wall area according to smoking status

|  | **Never-smokers** | | |  | **Current-Smokers** | | | **p-value** |
| --- | --- | --- | --- | --- | --- | --- | --- | --- |
|  | mean | SD | n= |  | mean | SD | n= |  |
| AWT @3.5 mm | 28.7 | 8.2 | 33 |  | 31.1 | 6.3 | 25 | 0.230 |
| AWT @4 mm | 33.2 | 7.8 | 46 |  | 36.1 | 6.8 | 50 | 0.055 |
| AWT @4.5 mm | 37.0 | 7.8 | 48 |  | 40.2 | 7.0 | 51 | 0.030 |
| AWT @5 mm | 40.2 | 8.0 | 48 |  | 43.6 | 7.0 | 51 | 0.023 |
| AWT @6 mm | 43.3 | 7.2 | 48 |  | 46.8 | 6.5 | 51 | 0.013 |
| AWT @8 mm | 46.9 | 7.0 | 48 |  | 49.6 | 5.9 | 50 | 0.041 |
| AWT @10 mm | 47.6 | 6.5 | 47 |  | 49.1 | 5.4 | 47 | 0.243 |

%AWA = airway wall area percentage; SD = standard deviation.

**Table S6:** Associations between airway wall thickness and age, gender and smoking status

|  | **Age** | |  | **Gender** | |  | **Smoking Status** | |
| --- | --- | --- | --- | --- | --- | --- | --- | --- |
|  | **b** | **p-value** |  | **b** | **p-value** |  | **b** | **p-value** |
| AWT @3.5 mm | -0.002 | <0.001 |  | 0.013 | 0.497 |  | 0.029 | 0.129 |
| AWT @4 mm | -0.003 | <0.001 |  | 0.008 | 0.639 |  | 0.032 | 0.056 |
| AWT @4.5 mm | -0.003 | <0.001 |  | 0.004 | 0.833 |  | 0.043 | 0.028 |
| AWT @5 mm | -0.003 | <0.001 |  | 0.003 | 0.891 |  | 0.051 | 0.027 |
| AWT @6 mm | -0.003 | <0.001 |  | 0.027 | 0.319 |  | 0.065 | 0.013 |
| AWT @8 mm | -0.003 | 0.005 |  | 0.017 | 0.652 |  | 0.067 | 0.059 |
| AWT @10 mm | -0.001 | 0.615 |  | -0.031 | 0.498 |  | 0.050 | 0.258 |

AWT = airway wall thickness; b = unstandardized regression coefficient.

**Table S7:** Associations between airway wall area and age, gender and smoking status

|  | **Age** | |  | **Gender** | |  | **Smoking Status** | |
| --- | --- | --- | --- | --- | --- | --- | --- | --- |
|  | **b** | **p-value** |  | **b** | **p-value** |  | **b** | **p-value** |
| %AWA @3.5 mm | -0.213 | <0.001 |  | 1.482 | 0.419 |  | 2.623 | 0.137 |
| %AWA @4 mm | -0.215 | <0.001 |  | 0.686 | 0.615 |  | 2.422 | 0.066 |
| %AWA @4.5 mm | -0.214 | <0.001 |  | 0.408 | 0.764 |  | 2.924 | 0.026 |
| %AWA @5 mm | -0.207 | <0.001 |  | 0.306 | 0.828 |  | 3.158 | 0.021 |
| %AWA @6 mm | -0.156 | <0.001 |  | 1.412 | 0.286 |  | 3.219 | 0.012 |
| %AWA @8 mm | -0.113 | 0.005 |  | 0.579 | 0.661 |  | 2.558 | 0.045 |
| %AWA @10 mm | -0.023 | 0.556 |  | -0.887 | 0.498 |  | 1.434 | 0.251 |

%AWA = airway wall area percentage; b = unstandardized regression coefficient.

**Table S8:** Association between pulmonary function parameters and airway wall thickness, independent of age, gender, smoking status and height

|  | **FEV_1_** | |  | **FEF_25-75_** | |  | **FVC** | |  | **FEV_1_/FVC** | |  | **RV/TLC** | |
| --- | --- | --- | --- | --- | --- | --- | --- | --- | --- | --- | --- | --- | --- | --- |
|  | b | p-value |  | b | p-value |  | b | p-value |  | b | p-value |  | b | p-value |
| AWT @3.5 mm | -0.131 | 0.095 |  | -0.219 | 0.100 |  | -0.126 | 0.187 |  | -0.796 | 0.271 |  | 0.522 | 0.403 |
| AWT @4 mm | -0.129 | 0.016 |  | -0.274 | 0.004 |  | -0.078 | 0.240 |  | -1.293 | 0.024 |  | 0.475 | 0.296 |
| AWT @4.5 mm | -0.145 | 0.005 |  | -0.292 | 0.002 |  | -0.093 | 0.149 |  | -1.408 | 0.011 |  | 0.242 | 0.585 |
| AWT @5 mm | -0.141 | 0.005 |  | -0.284 | 0.002 |  | -0.087 | 0.168 |  | -1.357 | 0.013 |  | 0.217 | 0.616 |
| AWT @6 mm | -0.142 | 0.005 |  | -0.284 | 0.002 |  | -0.098 | 0.120 |  | -1.357 | 0.040 |  | 0.180 | 0.674 |
| AWT @8 mm | -0.120 | 0.013 |  | -0.149 | 0.089 |  | -0.105 | 0.077 |  | -1.130 | 0.332 |  | 0.054 | 0.895 |
| AWT @10 mm | -0.038 | 0.410 |  | -0.043 | 0.605 |  | -0.038 | 0.498 |  | -0.511 | 0.873 |  | -0.106 | 0.785 |

|  | **TLCOc/VA** | |  | **R5** | |  | **R20** | |  | **R5-20** | |  | **X5** | |
| --- | --- | --- | --- | --- | --- | --- | --- | --- | --- | --- | --- | --- | --- | --- |
|  | b | p-value |  | b | p-value |  | b | p-value |  | b | p-value |  | b | p-value |
| AWT @3.5 mm | 0.026 | 0.284 |  | 0.017 | 0.150 |  | 0.012 | 0.177 |  | 0.005 | 0.433 |  | -0.002 | 0.665 |
| AWT @4 mm | 0.045 | 0.022 |  | 0.034 | 0.001 |  | 0.024 | 0.004 |  | 0.010 | 0.066 |  | -0.010 | 0.009 |
| AWT @4.5 mm | 0.039 | 0.046 |  | 0.033 | 0.001 |  | 0.026 | 0.001 |  | 0.007 | 0.207 |  | -0.007 | 0.047 |
| AWT @5 mm | 0.041 | 0.031 |  | 0.036 | <0.001 |  | 0.027 | 0.001 |  | 0.009 | 0.106 |  | -0.008 | 0.020 |
| AWT @6 mm | 0.036 | 0.064 |  | 0.033 | 0.001 |  | 0.026 | 0.001 |  | 0.007 | 0.170 |  | -0.009 | 0.007 |
| AWT @8 mm | 0.060 | 0.001 |  | 0.029 | 0.002 |  | 0.018 | 0.018 |  | 0.012 | 0.018 |  | -0.009 | 0.005 |
| AWT @10 mm | 0.034 | 0.048 |  | 0.020 | 0.027 |  | 0.014 | 0.049 |  | 0.006 | 0.232 |  | -0.005 | 0.177 |

Linear regressions with pulmonary function parameters as outcome and normalized airway wall thickness at different external diameters as predictor variable and age, gender, smoking status and height added as covariates; AWT = airway wall thickness; FEV_1_ = forced expiratory volume in 1 second; FVC = forced vital capacity; FEF_25-75_ = forced expiratory flow between 25-75% of FVC; RV/TLC = residual volume / total lung capacity; TLCOc = transfer capacity of the lung for carbon monoxide, corrected for hemoglobin level; VA = alveolar volume; R5 = resistance at 5 Hz; R20= resistance at 20Hz; R5-20 = difference between R5 and R20; X5 = reactance at 5 Hz; AX = reactance area, b = regression coefficient, it represents the change per SD change in predictor variable..

**Table S9:** Association between pulmonary function parameters and airway wall area, independent of

age, gender, smoking status and height

|  | **FEV_1_** | |  | **FEF_25-75_** | |  | **FVC** | |  | **FEV_1_/FVC** | |  | **RV/TLC** | |
| --- | --- | --- | --- | --- | --- | --- | --- | --- | --- | --- | --- | --- | --- | --- |
|  | b | p-value |  | b | p-value |  | b | p-value |  | b | p-value |  | b | p-value |
| %AWA @3.5 mm | -0.134 | 0.086 |  | -0.238 | 0.073 |  | -0.122 | 0.203 |  | -0.078 | 0.193 |  | 0.470 | 0.450 |
| %AWA @4 mm | -0.140 | 0.009 |  | -0.286 | 0.003 |  | -0.092 | 0.164 |  | -0.938 | 0.024 |  | 0.494 | 0.276 |
| %AWA @4.5 mm | -0.151 | 0.004 |  | -0.304 | 0.001 |  | -0.099 | 0.126 |  | -1.296 | 0.011 |  | 0.249 | 0.576 |
| %AWA @5 mm | -0.149 | 0.003 |  | -0.295 | 0.001 |  | -0.097 | 0.127 |  | -1.427 | 0.013 |  | 0.215 | 0.623 |
| %AWA @6 mm | -0.149 | 0.003 |  | -0.294 | 0.001 |  | -0.106 | 0.095 |  | -1.376 | 0.034 |  | 0.203 | 0.636 |
| %AWA @8 mm | -0.116 | 0.017 |  | -0.147 | 0.096 |  | -0.102 | 0.089 |  | -1.173 | 0.348 |  | 0.021 | 0.959 |
| %AWA @10 mm | -0.037 | 0.248 |  | -0.042 | 0.615 |  | -0.035 | 0.534 |  | -0.496 | .0844 |  | -0.108 | 0.782 |

|  | **TLCOc/VA** | |  | **R5** | |  | **R20** | |  | **R5-20** | |  | **X5** | |
| --- | --- | --- | --- | --- | --- | --- | --- | --- | --- | --- | --- | --- | --- | --- |
|  | b | p-value |  | b | p-value |  | b | p-value |  | b | p-value |  | b | p-value |
| %AWA @3.5 mm | 0.025 | 0.312 |  | 0.020 | 0.088 |  | 0.013 | 0.125 |  | 0.006 | 0.309 |  | -0.004 | 0.508 |
| %AWA @4 mm | 0.047 | 0.017 |  | 0.035 | 0.001 |  | 0.025 | 0.002 |  | 0.010 | 0.063 |  | -0.010 | 0.007 |
| %AWA @4.5 mm | 0.040 | 0.043 |  | 0.033 | 0.001 |  | 0.026 | 0.001 |  | 0.007 | 0.188 |  | -0.007 | 0.048 |
| %AWA @5 mm | 0.042 | 0.030 |  | 0.036 | <0.001 |  | 0.027 | 0.001 |  | 0.009 | 0.103 |  | -0.008 | 0.021 |
| %AWA @6 mm | 0.036 | 0.060 |  | 0.034 | 0.001 |  | 0.027 | 0.001 |  | 0.007 | 0.167 |  | -0.009 | 0.008 |
| %AWA @8 mm | 0.060 | 0.001 |  | 0.029 | 0.002 |  | 0.017 | 0.021 |  | 0.011 | 0.022 |  | -0.009 | 0.006 |
| %AWA @10 mm | 0.033 | 0.057 |  | 0.021 | 0.023 |  | 0.015 | 0.042 |  | 0.006 | 0.235 |  | -0.005 | 0.152 |

Linear regressions with pulmonary function parameters as outcome and normalized airway wall thickness at different external diameters as predictor variable and age, gender, smoking status and height added as covariates; %AWA = airway wall area percentage; FEV_1_ = forced expiratory volume in 1 second; FVC = forced vital capacity; FEF_25-75_ = forced expiratory flow between 25-75% of FVC; RV/TLC = residual volume / total lung capacity; TLCOc = transfer capacity of the lung for carbon monoxide, corrected for hemoglobin level; VA = alveolar volume; R5 = resistance at 5 Hz; R20= resistance at 20Hz; R5-20 = difference between R5 and R20; X5 = reactance at 5 Hz; AX = reactance area, b = regression coefficient, it represents the change per SD change in predictor variable.
