# Supplementary figures and images for: Airway wall thickness on HRCT scans decreases with age and increases with smoking
Source: BMC Pulm Med. 2017 Feb 1;17:27. doi: 10.1186/s12890-017-0363-0 (PMC5286807; doi:10.1186/s12890-017-0363-0)

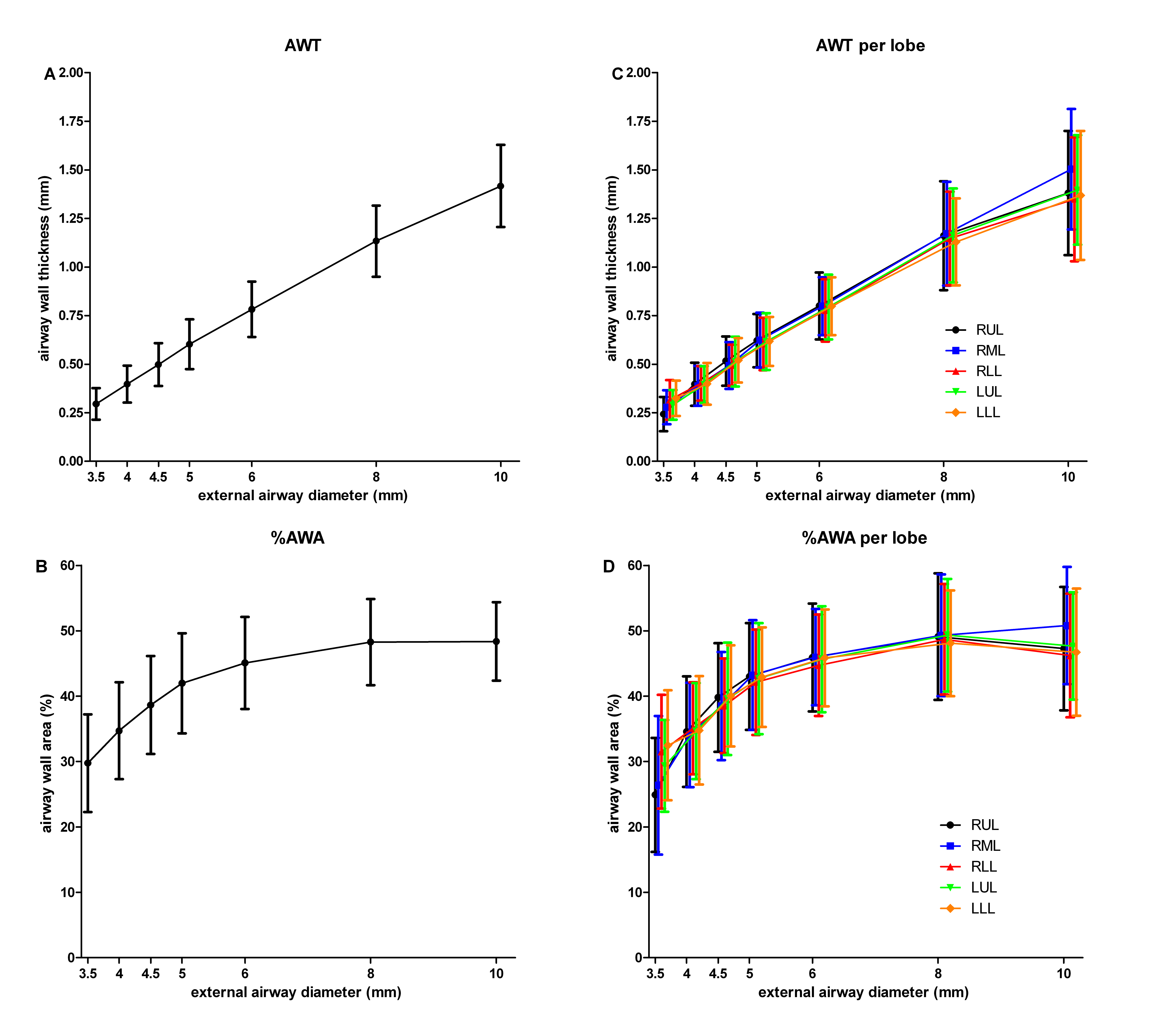

Supplement: Additional file 2: Figure S1. — AWT and %AWA at different external airway diameters. AWT = airway wall thickness; %AWA = airway wall area percentage. (TIF 3044 kb) [file 12890_2017_363_MOESM2_ESM.tif]
